# Photobiomodulation, Compared to Revascularisation, and Conservative Treatment—What Works for Healing Hard‐to‐Heal Arterial Leg Ulcers in Older Adults: A Quasi‐Experimental Study

**DOI:** 10.1111/wrr.70106

**Published:** 2025-11-11

**Authors:** Marianne Degerman, Laura Corneliusson, Micael Öhman, Marcus Schmitt‐Egenolf, Bo Christer Bertilson, Åsa Audulv

**Affiliations:** ^1^ Department of Nursing Umeå University Umeå Sweden; ^2^ Department of Development Skellefteå Municipality Skellefteå Sweden; ^3^ Division of Wood Science and Engineering, Department of Engineering Sciences and Mathematics Luleå University of Technology Luleå Sweden; ^4^ Department of Public Health and Clinical Medicine, Dermatology Umeå University Umeå Sweden; ^5^ Division of Family Medicine and Primary Care, Department of Neurobiology, Care Sciences and Society Karolinska Institutet Stockholm Sweden

**Keywords:** arterial leg ulcer, older adults, photobiomodulation, quasi‐experimental study, wound treatment

## Abstract

Hard‐to‐heal arterial leg ulcers in older adults are a challenging and complex condition. In this quasi‐experimental study, three treatment approaches were compared. The purpose was to investigate (1) the healing time of arterial leg ulcers in older adults (≥ 70 years) who underwent photobiomodulation, revascularisation, or conservative treatment; (2) the importance of factors associated with impaired healing; and (3) ulcer recurrence after healing with photobiomodulation. Participants who received photobiomodulation (*n* = 51) were frail older adults recruited from municipal home healthcare and matched with participants who received revascularisation (*n* = 71) or conservative treatment (*n* = 153). The latter two groups were retrieved from the Swedish Quality Registry RiksSår for ulcer treatment. Photobiomodulation was performed at wavelengths of 635 and 904 nm twice weekly. The results showed that the photobiomodulation group had a significantly shorter healing time (*p* < 0.001) and a higher proportion of healed ulcers; photobiomodulation 66.7%, revascularized 50.7% and conservatively treated group 41.2%. The median healing times for the photobiomodulation group were 135 days (confidence interval 95–175), compared to 252 (confidence interval 181–323) and 316 (confidence interval 192–440) in revascularized and conservatively treated groups, respectively. Neither ulcer duration nor other pretreatment factors exerted clinically relevant effects on healing time. In this study, recurrence within 24 months of healing with photobiomodulation was < 12%. In conclusion, photobiomodulation has the potential to heal hard‐to‐heal arterial ulcers markedly faster than revascularisation or conservative treatment. It could be a suitable treatment alternative for frail older adults, including those with previous substantial ulcer duration.

**Abbreviations**
ALU: arterial leg ulcer
ASC group: arterial surgery control group
CI: confidence interval
CTC group: conservatively treated control group
ECM: extracellular matrix
GaAllnp: heliumneon
GaAs: galliumarsenid
HR: hazard ratio
Hz: hertz
IQR: interquartile range
J: joule
mW: milliWatt
Nm: nanometer
PBM: photobiomodulation
PBM group: photobiomodulation group
RCT: randomised clinical trial
SD: standard deviation
SPSS: Statistical Package for the Social Sciences

## 1 Introduction

Arterial leg ulcers (ALU) account for 5%–20% of all hard‐to‐heal ulcers [1]. ALUs represent the highest increased risk of amputation and mortality among the different aetiologies of hard‐to‐heal ulcers especially in older adults [2, 3]. In addition to the clinical burden, ALU severely diminishes the individual's quality of life, contributing to pain [1, 4], mobility impairment [1], and sleep disturbances [1, 4]. Treatment and complications are associated with considerable healthcare costs, with ALUs ranking among the most expensive ulcer types to treat, especially in outpatient settings [5, 6].

The pathophysiology of ALU involves insufficient arterial blood perfusion to lower extremity tissues, leading to inadequate nourishment, tissue breakdown, and ulcer formation [1, 3, 7]. ALUs are diagnosed by clinical assessment, diminished tibial or pedal pulses, and an ankle‐brachial systolic blood pressure index of < 0.9 [3, 8]. When ALUs fail to progress healing within 4–6 weeks, they are classified as hard‐to‐heal [9]. In hard‐to‐heal ALUs, healing is complicated by both local factors, for example, infection, hypoxia, necrotic tissue, ulcer area [10], ulcer duration, arterial sufficiency and systemic factors, for example, comorbid conditions, ambulatory status and advanced age [1, 10]. Thus, older adults are particularly vulnerable compared to middle‐aged or younger adults, yet they are frequently excluded from clinical trials, limiting the generalisability of treatment efficacy in that population [11]. This emphasises the importance of individualised holistic assessment strategies and the development of new treatment approaches [1, 12, 13, 14].

Current standard‐of‐care treatment for ALUs includes revascularisation, physical activity, ulcer dressings and debridement [9, 13, 15]. Revascularisation and physical activity aim to restore perfusion and promote tissue repair and are therefore considered to be the best treatment, option [9, 13, 15]. Adapted ulcer dressings and debridement are often combined with revascularisation [9, 13]. Still, they can also be performed as a stand‐alone treatment defined as conservative treatment, in cases when revascularisation is not suitable [1, 9, 13]. Older adults are often deemed ineligible for invasive revascularisation and may have limited ambulatory status, reducing their treatment options [1, 13]. Even after successful arterial revascularisation, healing outcomes vary remarkably, with a 62%–91% healing rate within 1 year, with median healing times between 56 and 570 days [16]. The recurrence rate of ALUs is high, especially after healing with a conservative treatment approach; within 3 months after healing, recurrence is up to 43% [17].

Laser photobiomodulation (PBM) is a relatively new approach to treating hard‐to‐heal ulcers. PBM has been shown to have the potential to improve wound healing [18, 19, 20, 21, 22, 23, 24, 25]; PBM can reduce inflammation, pain, and oedema and also support the regeneration of damaged tissues [19]. PBM is a non‐invasive light therapy with a nonthermal process in tissues [20]. Studies on PBM at the tissue and cell levels have identified the induction of a photochemical reaction, which affects three of the four wound healing phases: the inflammatory, proliferative, and remodelling phases, facilitating more rapid wound closure [18, 19, 20, 21, 22, 23, 24, 25]. In addition, there are no known adverse effects associated with PBM [26, 27, 28, 29]. Clinical studies have documented the potential of PBM in healing diabetic foot ulcers, including a reduction in ulcer size [23, 26, 27, 29]. However, multiple factors, such as neuropathic and/or metabolic, are involved in diabetic foot ulcer healing that are inapplicable to ALUs in patients without diabetes [1, 12, 30]. Few studies on PBM have specifically addressed ALUs. One clinical study compared microcirculation at the ulcer edge and wound bed score between patients with diabetes and peripheral arterial disease and those with ALU without diabetes and controls [28]. During an 8‐week trial, both PBM‐treated groups showed substantially increased microcirculation and granulation in the wound bed after thrice‐weekly treatment [28].

Currently, available evidence on ALU and PBM treatment remains scarce; published studies have short intervention and follow‐up periods and exclude older adults. Since older adults are less likely to be eligible for revascularisation, other non‐invasive treatment options could be especially beneficial to them.

The current study aims to investigate the healing of ALUs in older adults who have received PBM, in comparison to revascularisation and conservative treatment. The following research questions were examined:

1. Does PBM reduce the healing time of ALU compared to (i) revascularisation and (ii) conservative standard‐of‐care?
2. Do factors such as ulcer duration, age, sex, ulcer area, ambulatory status, and number of comorbid conditions affect the healing time with either type of treatment?
3. What is the recurrence rate of ALU at 6, 12, 18, and 24 months after healing with PBM?

## 2 Materials and Methods

### 2.1 Study Design and Ethical Statement

This quasi‐experimental study compared a prospective intervention cohort of frail older adults receiving PBM treatment for hard‐to‐heal ALUs (PBM group) with (i) a control group of patients receiving arterial surgery (ASC group) and (ii) a conservatively treated control group (CTC group). The PBM group was recruited from patients receiving treatment for hard‐to‐heal ALU by primary home healthcare in the municipality of Skellefteå between 2019 and 2022. The ASC and CTC groups were selected from retrospective data in the National Swedish Quality Registry for ulcer treatment RiksSår [31]. Data were retrieved from RiksSår on 22 October 2020. Inclusion criteria for all groups were patients aged ≥ 70 years with hard‐to‐heal ALUs diagnosed by a physician. Hard‐to‐heal ALU in this study was defined as an ulcer duration of ≥ 6 weeks. Patients aged ≥ 70 years were selected, as home healthcare populations predominantly consist of older adults, with few individuals below 70 years of age. The CTC group was matched with the PBM group using the variable of ulcer duration at baseline. The ASC group consisted of all eligible cases in the registry that fulfilled the inclusion criteria and had received revascularisation.

Ethical approval was obtained from the Swedish Ethical Review Authority in Lund, Sweden (Dnr. 2020‐02194) and further complemented in 2024 (Dnr. 2024‐00844‐02). Written informed consent was obtained from the participants and/or legal guardians in cases of impaired cognition in the PBM group. A consent procedure is applied for registration in the RiksSår Registry. The study protocol adhered to the ethical guidelines of the 2024 Declaration of Helsinki [32].

### 2.2 Data Material

Baseline data included demographic variables: age, sex, number of comorbid conditions, presence of diabetes, ulcer duration, ulcer area (length × width in cm^2^), and ambulatory status. Ambulatory status was rated by a nurse in the following categories: bedbound, wheelchair user, walking with assistance, and walking. Outcome data included data regarding arterial surgery during registration time (yes/no) and treatment events: healed ulcer (completely closed ulcer/intact skin), amputation, death, and interrupted treatment (patient changing clinic or discontinuation of treatment). The main outcome was the time to healing in days.

Participants in the three groups were followed two to three times weekly until a treatment event occurred or for up to 1000 days.

To determine the recurrence of ALUs among participants in the PBM group after ulcer healing was achieved, the status of healed ulcers was extracted from patient records at 6, 12, 18, and 24 months. The definition of recurrence was ALU within the prior ulcer location.

### 2.3 Study Procedure and Selection Criteria

The PBM group received treatment in a project initiated by Skellefteå municipality in northern Sweden. In the project, frail older adults were offered PBM to explore the value of additional treatment of hard‐to‐heal ALUs in domestic home healthcare. Treatment was conducted by trained healthcare providers in the patients' homes/nursing homes.

Nurses working in domestic home healthcare and nursing homes recruited eligible patients in collaboration with general practitioners. All eligible and consenting patients were included.

Before PBM, it was ensured that frail older adults with ALU were treated according to home healthcare's current routine. This included an adequate ulcer treatment plan and regular risk assessments using the Senior Alert quality register [33] to identify risks of malnutrition, falls, deteriorating oral health and pressure ulcers.

**FIGURE 1:**
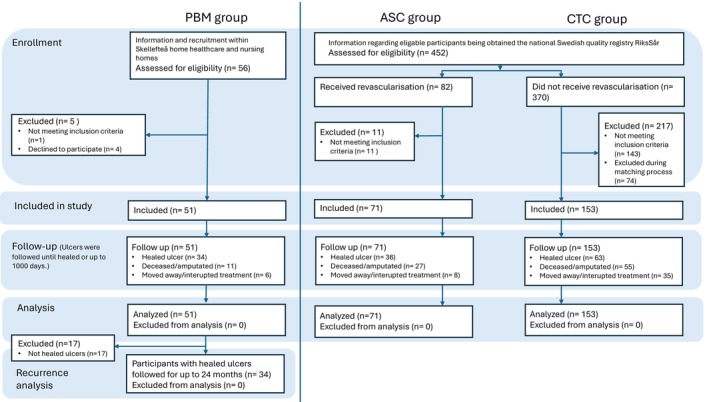
Enrolment of participants in the PBM (photobiomodulation) group, the ASC (arterial surgery control) group, and the CTC (conservatively treated control) group. *n* = number of participants.

The CTC and ASC groups were recruited from the Swedish registry RiksSår (established in 2009). In 2020, the national coverage for RiksSår was approximately 20% of ulcer treatment facilities, with over 20,000 registrations of different aetiologies recorded. Of these, municipality primary home healthcare represented 22% of the registrations, primary healthcare centres represented 43%, and specialist units in hospital care accounted for 35%, respectively. All registrations in RiksSår are conducted by the nurse responsible for the ulcer treatment [31]. Figure 1 illustrates the enrolment process.

### 2.4 Treatment Procedure

Each ALU received standard‐of‐care treatment based on national guidelines for ALU treatment [9]. Treatment was provided two to three times weekly according to the physicians' prescription. The CTC group received conservative treatment (e.g., debridement and ulcer dressing according to guidelines) [9]. The ASC group received a revascularisation procedure in addition to debridement and ulcer dressing [9]. The PBM group received debridement, ulcer dressing, and additional PBM treatment.

### 2.5 PBM Treatment

The PBM treatment procedure and dosing parameters were based on the World Association for Photobiomodulation Therapy (WALT) recommendations for inflammation [34] and clinical experience with the patient group. PBM treatment frequency was determined to be twice weekly, with an interval of 3–4 days, limiting the ALU exposure to environmental temperatures, which negatively affects ulcer healing [9], yet ensuring PBM treatment efficacy. PBM equipment was Mid‐Laser (Spectro Analytic Irradia AB, Fagerstagatan 9, 163 53 Spånga, Sweden). The characteristics and technical specifications of the equipment are described in Table 1. The dose emitted from handpieces was calibrated following the manufacturer's instructions [35] before the study, every 6 months during the study, and after the completion of the study. The treatment team consisted of four healthcare‐trained ulcer treatment specialists who had undergone supervised PBM training. The PBM procedure included infrared wavelength exposure to lymphatic areas and ulcer areas, aiming to enhance immune and circulatory systems often compromised in this patient group, and red wavelength PBM, known to affect the ulcer healing phases. The device was managed manually during treatment. The treatment time varied between 20 and 40 min, depending on ulcer size and dressing complexity. The dressing complexity was influenced by factors such as large ulcers requiring multiple dressings for coverage, heavy exudate necessitating layered dressings to prevent ulcer edge maceration, or ulcers located in anatomically challenging areas, such as the tips of the toes.

**TABLE 1:** Laser photobiomodulation characteristics.

Laser‐type handpiece	12 GaAs laser diodes	4 GaAllnp laser diodes
Wavelength	904 nm	635 nm
Power output	60 mW	75 mW
Peak power output	25,000 mW	
Pulse frequency	700 Hz	250 Hz
Beam	Divergent	Divergent
Aperture diameter in contact application	5 mm	9 mm

*Note:* The technical specifications refer to each laser diode.

Infrared 904 nm, 60 mW PBM targeted the lymphatic and ulcer areas; a fluence of 7.2 J/cm^2^ was applied bilaterally to the shoulder/neckline for 120 s, and the same fluence was applied to the hollow of the knee for 120 s. Intact skin close to the ALU was treated for 30 s per location above, below, and on each side at a fluence of 1.8 J/cm^2^ using the contact application technique. The ALU surface was treated at a distance of 1 cm, using a projection application technique for 120 s per position, with a dose of 7.2 J emitted per laser diode until the total surface was treated. Red 635 nm, 75 mW PBM targeted ulcer edges and surface: ulcer edges: a fluence of 2.25 J/cm^2^ was applied for 30 s, stepwise moved 1 cm between positions until the total ulcer edge was treated, using the contact application technique. The ALU surface was treated at a distance of 1 cm, using a projection application technique for 120 s per position, with a dose of 9 J emitted from the laser diode until the entire surface was treated.

### 2.6 Statistical Analysis

A data matching procedure of three to one was performed for selecting the CTC group; three ALUs from RiksSår were matched to one PBM ALU. Matching was based on the ALU duration at baseline. When an absolute match was not detected, the nearest match was selected; random selection was applied when more than three ALUs matched. One‐way ANOVA with Welch's correction was used to determine significance *p* values at baseline. Descriptive statistics, such as percentages and standard deviations (SDs), were employed to present the demographics of the three groups and the recurrence rate in the PBM group.

Survival analysis was used to calculate the time to ulcer healing, which was performed using the Kaplan–Meier method. The following treatment events were censored: amputation, death and interrupted treatment. Differences between groups were analysed using the log rank test. Hazard ratios (HRs) and corresponding 95% confidence intervals (CIs), using the Cox proportional hazards model, were calculated to determine the impact of different factors on healing time during treatment. The model included age, sex, ulcer area, ambulatory status, number of comorbid conditions, ulcer duration, and treatment. All alpha levels were set to 5%, two‐sided. Statistical analysis was performed with the Statistical Package for the Social Sciences software program SPSS version 29.0 (IBM Corp., Armonk, NY, USA) [36].

## 3 Results

### 3.1 Patient Characteristics

Overall, the ASC group had the highest health status, followed by the CTC group, with the PBM group demonstrating the lowest health status among the groups (Table 2). Patients in the ASC group were younger, had shorter ulcer duration, and had fewer comorbid conditions. In contrast, patients in the PBM group had more comorbid conditions (*p* < 0.001) along with the highest incidence of diabetes. The median ulcer areas were similar among the three groups. Regarding ambulatory status, the PBM group had the lowest status (*p* < 0.001), with 56.9% of patients using a wheelchair and only 37% having walking ability. In the ASC group and CTC group, 11.3% and 15% of patients used wheelchairs, respectively, whilst over 75% of patients in these groups could walk. The sex distribution differed between the groups; the PBM and the ASC groups had a majority of males, 54.9% and 53.5%, respectively, whereas the CTC group had a majority of females, 57.5%.

**TABLE 2:** Baseline characteristics of patients in the respective treatment groups.

Patients' characteristics	PBMG, *N* = 51	ASCG, *N* = 71	CTCG, *N* = 153
Age in years, mean/SD	83.2/7.0	80.8/7.2	83.3/7.2
Male, *N*/%	28/54.9	38/53.5	65/42.5
Female, *N*/%	23/45.1	33/46.5	88/57.5
Ulcer area in cm^2^, mean/SD	11.5/28.9	12.8/32.1	12.1/25.4
Ulcer area in cm^2^, median/IQR	4.0/2.3–9.0	2.9/1.0–8.4	3.0/0.8–10.0
Ulcer duration in weeks. mean/SD	28.8/35.5	22.7/38.6	28.7/33.4
Ulcer duration in weeks, median/IQR	16/7.0–31.0	11/6.0–20.0	16/8.0–35.0
Number of conditions, mean/SD	2.5/0.9*	1.6/0.8	1.7/0.9
Diabetes, *N*/%	28/54.9	26/36.6	73/47.7
Ambulatory status	**		
Bedbound, *N*/%	0/0.0	2/2.8	1/0.7
Wheelchair user, *N*/%	29/56.9	8/11.3	23/15
Walking with assistance, *N*/%	3/5.9	6/8.5	13/8.5
Walking, *N*/%	19/37.3	55/77.5	116/75.8

Abbreviations: ASCG, arterial surgery control group; CTCG, conservatively treated control group; IQR, interquartile range; PBMG, photobiomodulation group; SD, standard deviation.

* Statistically significant difference between PBMG in the mean number of conditions and ASCG and CTCG, *p* < 0.001.

** Statistically significant difference between PBMG ambulatory status and ASCG and CTCG *p* < 0.001.

### 3.2 Treatment Outcomes

The PBM group had the highest percentage of healed ALUs, with 66.7% (*n* = 34), followed by the ASC group (50.7%; *n* = 36) and CTC group (41.2%; *n* = 63). In the PBM group, 33.3% (*n* = 17) of ALUs were censored. ALUs were censored owing to the following reasons: (1) amputation not related to ALUs (*n* = 2); (2) death (*n* = 9); (3) hospital admission unrelated to ALUs (*n* = 2); (4) treatment disruption due to physicians' prescription or isolation guidelines for the COVID–2019 pandemic (*n* = 3); and (5) treatment disruption due to the limited effect of PBM after 6 weeks of treatment (*n* = 1). All censored ALUs in the PBM group, except one, exhibited signs of healing until censoring.

In addition, 49.3% (*n* = 35) and 58.8% (*n* = 90) of ALUs were censored in the ASC group and CTC group, respectively, owing to amputation, death, or treatment interruption.

### 3.3 Healing Time

**FIGURE 2:**
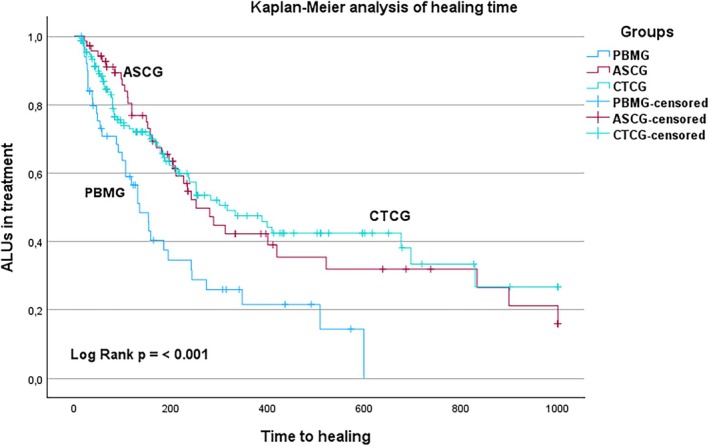
Kaplan–Meier analysis of healing time. *Y*‐axis: ALU (arterial leg ulcer) in treatment, 100%–0%. *X*‐axis: Time in days to healing, limited to 1000 days. ASCG, arterial surgery control group; CTCG, conservatively treated control group, censored: when another event other than healing occurred (amputation, death, or treatment interruption); PBMG, photobiomodulation group.

The PBM group had a significantly shorter healing time than the ASC group and CTC group (*p* < 0.001) (Table 3 and Figure 2). The PBM group had a median healing time of 135 days (95% CI, 95–175 days). In contrast, the ASC group had a median healing time of 252 days (95% CI, 181–323 days). The CTC group had the longest healing time with a median of 316 days (95% CI, 192–440 days).

**TABLE 3:** Time to healing in days for each treatment group.

Group healed ALUs, *n*/*N* (%)	Mean, SE_mean_	95% CL_mean_	Median, SE_median_	95% CL_median_
PBMG, 34/51 (66.7)	218, 33	154–283	135, 21	95–175
ASCG, 36/71 (50.7)	446, 56	336–556	252, 36	181–323
CTCG, 63/153 (41.2)	478, 43	394–563	316, 63	192–440

Abbreviations: %, percentage of healed ulcers in the respective groups; ASCG, arterial surgery control group; CI, confidence intervals; CTCG, conservatively treated control group; Healed ALU *n*, number of healed arterial ulcers; *N*, total number of ulcers per group; PBMG, photobiomodulation group; SE, standard error.

### 3.4 Impact of Different Factors on Healing Time During Treatment

Ulcer duration, age, ambulatory status, and the number of comorbid conditions were not associated with ulcer healing time based on the Cox proportional hazards model. The model revealed that the ulcer area contributed negatively to healing time, with an HR of 0.984 with CI 0.973–0.994, indicating a 1.6% increase in healing time per additional cm^2^ (*p* = 0.002). The ulcer area did not impact between‐group differences, since the ulcer area was similar across the groups. The female sex contributed positively to healing time, with an HR of 1.6 with CI 1.058–2.435, indicating a 60% reduction in healing time in females (*p* = 0.026). However, additional Kaplan–Meier analysis controlling for sex did not detect a significant difference in ALU healing time between sexes (*p* = 0.53).

### 3.5 Recurrence Rate of ALUs After Healing With PBM Treatment

**FIGURE 3:**
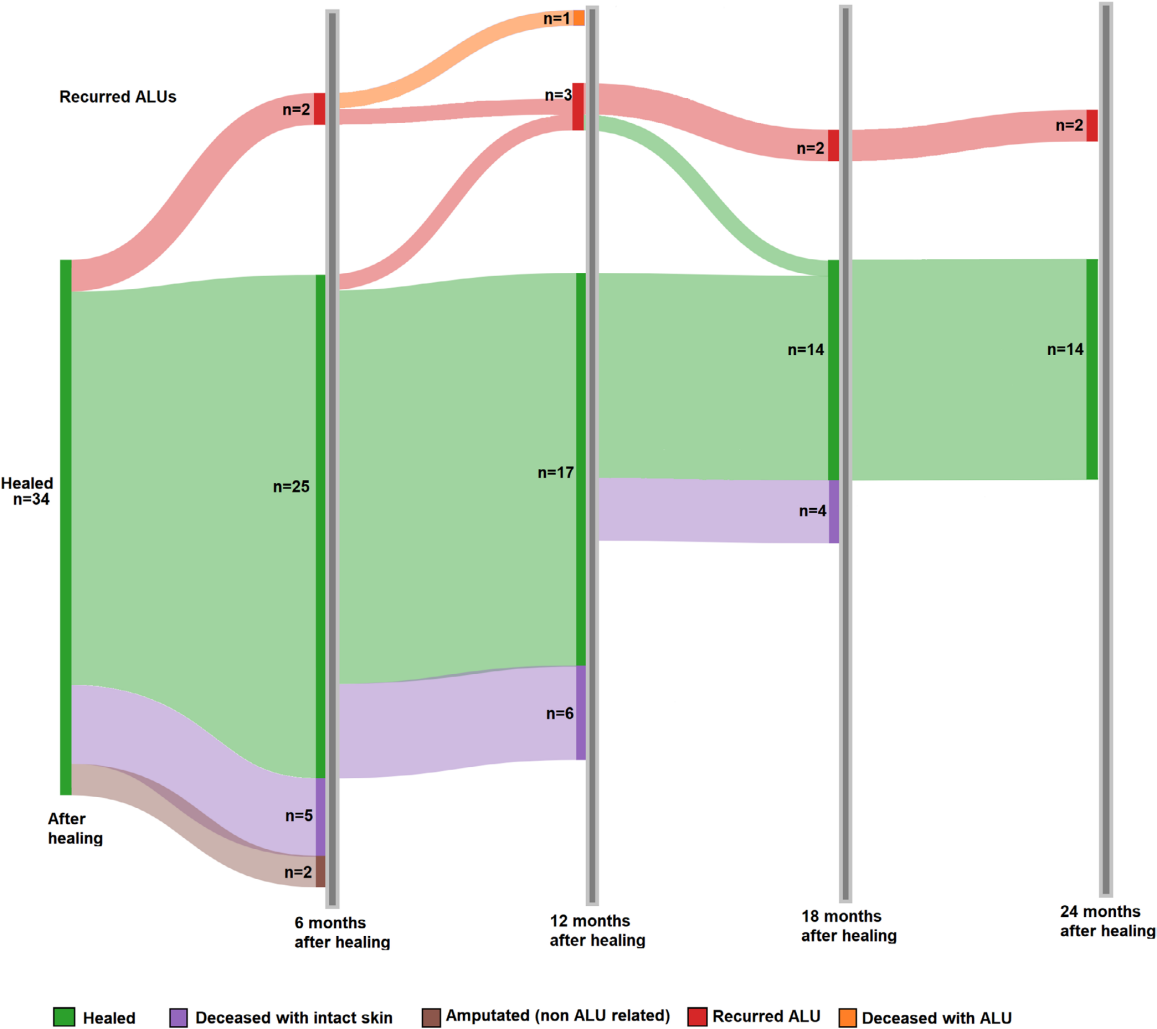
Healed ALU (arterial leg ulcer) after PBM (photobiomodulation) treatment and at 6, 12, 18, and 24 months of follow‐up. After 24 months, recurrence can be observed in less than 12% (*n* = 4) of ALUs.

After being healed, the patients in the PBM group were followed up at 6, 12, 18, and 24 months to determine the recurrence rate of ALUs (Figure 3). In total, 11.8% (*n* = 4) of the 34 ALUs recurred during the follow‐up period. After 24 months, 14 ALUs (41.2%) remained healed, whilst two had recurred and persisted during the follow‐up period; one patient had died with a recurred ALU, and 15 patients died with intact skin.

## 4 Discussion

In the current study, the PBM group exhibited a substantially shorter ALU healing time than the ASC and the CTC groups. This beneficial outcome was observed despite the PBM group having poorer health status before treatment; for example, more comorbid conditions, higher incidence of diabetes, and limited ambulatory status. The PBM group had a median healing time that was about 4 months faster than that of the ASC group and 6 months faster than that of the CTC group. Reducing ALU healing time by several months has the potential to decrease the risk for complications, such as systemic infection and pain, as well as positively impact quality of life. Also, healthcare‐associated costs may be reduced; for example, costs related to prolonged treatment and/or extensive caregiving. The health‐economic aspects of PBM treatment of ALUs are important to address in future research.

Previous research has described associations between ulcer healing and ulcer duration [11, 12, 14], indicating that the longer an ulcer persists, the less likely it is to heal. In the current study, no association was found between ulcer duration and healing time. Those findings indicate that PBM could be a promising treatment option also for patients who have had ALUs for several years, which is not the case with current standard‐of‐care treatments. PBM promotes ALU healing through multiple cellular mechanisms [18, 19, 20, 21, 22, 23, 24, 25]. This is in contrast to conservative treatment, which only affects the ulcer surface, and revascularisation, which is aimed at facilitating ulcer healing by increasing blood flow in the lower extremities [9, 13]. Whereas PBM stimulates cytochrome c oxidase in the mitochondrial respiratory chain, leading to increased adenosine triphosphate production, enhanced cell metabolism and improved energy availability for reparative processes [18, 19, 22]. Furthermore, PBM enhances angiogenesis by upregulating vascular endothelial growth factor and nitric oxide production, improving microcirculation and oxygen delivery in ischaemic or poorly perfused tissue [20, 21, 22]. PBM also modulates the inflammatory response by downregulating pro‐inflammatory cytokines and promoting a shift towards anti‐inflammatory signalling [21, 22].

Revascularisation procedures are currently considered to be the best for ALU treatment [9, 13]. Despite this, the ulcer healing time can still be prolonged after successful revascularisation, and complete healing 1 year post‐procedure varies between 60% and 90% [16]. However, some patient groups are not suitable for invasive treatments. Frailty, which is common among older adults receiving home healthcare, is an independent risk factor for postoperative complications [37]. After invasive treatments, frail older adults risk institutionalisation in nursing homes, facing increased dependency and/or mortality [37]. Therefore, frail older adults are omitted from the current best treatment for ALU. With today's demographic development, the number of frail older adults is increasing; therefore, timely and effective ulcer healing is especially critical for this group. In the present study, PBM was administered to patients with high levels of frailty, several of whom were homebound. Since PBM is a non‐invasive and non‐painful treatment [18, 19, 20, 21, 22, 23, 24, 25], it can be used to treat ALUs also for patients with frailty who are not eligible for invasive treatments. This indicates that PBM could be a valuable contribution to the toolbox of ALU treatments, especially for frail older adults.

In this study, the duration of the ALU treatment was not confined to a predetermined timeframe or number of treatment occasions. Most studies investigating PBM have a fixed number of treatment occasions, for example, a number of days with PBM or a number of weeks with PBM, and a follow‐up period of < 3 months [21, 23, 24, 26, 29]. In such cases, healing time cannot be determined; instead, usual outcomes include decreased ulcer area. This is problematic since hard‐to‐heal ALU healing is not guaranteed to proceed to complete healing even if the healing process is initiated. Factors such as infection and health status can interfere so that the ALU regresses to impaired healing. This study is unique since it follows the healing of ALUs in a clinical, real‐life setting for several years. Patients were followed until complete healing was achieved, and the recurrence rate post‐healing was followed for 2 years. To our knowledge, there are no other research studies conducted with even a comparative time frame. The recurrence rates observed in the current study are markedly lower, < 12% 2 years post‐PBM treatment, than those reported in previous studies that investigated conservative treatment with 43% 3 months post‐healing [17]. Recurrence can be expected in patients during the terminal process, owing to the deteriorating health condition of the dying body, with tissue breakdown as a natural consequence. Several frail older adults in the PBM group died during the follow‐up period. Although recurrence could be expected in the patients who were in terminal status, ALU recurrence was observed in only 1 of 16 patients during the terminal process. This might be explained by the PBM treatment stimulating the extracellular matrix (ECM) [18, 22], which is often compromised in frail older adults [11]. ECM influences collagen synthesis and matrix metalloproteinase regulation, leading to improved ECM organisation and tensile strength of the repaired tissue [18, 19, 22]. This may, in addition to the PBM effect on angiogenesis and microcirculation that aid the healing of the ALU [20, 21, 22], also support the long‐term effects of the treatment [18, 19, 20, 21, 22].

Consistent with previous PBM studies [21, 22, 23, 24, 25, 26, 27, 28, 29], this study revealed that PBM positively impacted hard‐to‐heal ulcer healing. Furthermore, frail older adults have been investigated in an earlier study by this research team administering PBM on hard‐to‐heal venous leg ulcers, finding a 123‐day faster healing in the PBM group despite their frailty [38]. However, there is a lack of consensus regarding various key parameters, including the optimal power and wavelength for ulcer healing: red light, infrared light, or a combination of both [22, 26]. Previous studies ask for standardisation of the dose, frequency, and duration of treatment, as well as treatment applications, such as projection technique or skin contact [23]. In this study, a combination of red and infrared PBM wavelengths was employed because the treatment could then target both the lymphatic system and the local ALU area, eliciting both systemic and local therapeutic effects [18, 19, 20, 22]. Stimulating the lymphatic system facilitates the removal of inflammatory mediators, cellular debris, and excess interstitial fluid from the ALU, balancing the immune and circulatory systems [18, 19, 20], often compromised in frail older adults with ALUs. Furthermore, frail older adults' ulcer healing is complex owing to interacting issues in the healing process; the combination of deep infrared and more superficial red stimulation was used to enhance a multifaceted regenerative process [21, 22]. The treatment frequency, twice weekly, was chosen to minimise prolonged ALU exposure to ambient environmental temperatures, which are known to impede the healing process [9]. The treatment needed to have both a clinically effective treatment dose and be feasible to deliver by the home healthcare providers.

Randomised controlled trials (RCTs) are the gold standard for evidence‐based research, minimising the risk of bias. However, RCTs have also been criticised for their limited generalisability to complex healthcare environments [39, 40], showing limitations in external validity; for example, individuals with comorbid conditions and older adults are frequently excluded from RCTs, populations that are highly representative of real‐world clinical settings [11, 12, 40, 41]. Furthermore, RCTs occasionally involve extensive protocols that can be challenging and impractical to implement in clinical practice, particularly in home healthcare settings. For example, PBM studies that treat ALUs once a day for a short time period can provide important details on the healing process in tissue and short‐term patient effects such as decreased pain. However, daily treatments are not feasible to implement in the clinical practice of hard‐to‐heal ALUs, which often take months to heal [21, 26]. In this study, randomisation was not possible due to ethical and organisational constraints tied to the setting in which the PBM was delivered. Instead, a quasi‐experimental design was employed; the PBM group was compared to two control groups extracted from the RiksSår quality registry. The PBM group, ASC group, and CTC group showed demographic differences at baseline. However, the PBM group had higher rates of factors associated with impaired ulcer healing, ensuring that the effects of PBM were not overestimated. Quasi‐experimental designs are especially valuable in applied healthcare research where real‐world feasibility, patient accessibility, and treatment implementation are key priorities. By integrating data from the RiksSår registry reflecting typical clinical populations, this study offers a pragmatic assessment of PBM treatment effectiveness, enhancing its relevance to everyday clinical practice. Whilst causal interferences are inherently more limited in quasi‐experimental studies than in RCTs, the design employed allows for a meaningful evaluation of intervention outcomes under realistic conditions [42].

## 5 Limitations

However, there are also limitations. For the ASC group, the quality registry did not specify the type of procedure the patients underwent; therefore, a comparison of differences between types of revascularisation procedures was not possible. In addition, a limited number of patients in the RiksSår registry aged ≥ 70 years had undergone revascularisation, which rendered a matching procedure between the ASC and PBM groups impossible. The CTC group was matched against ALUs in the PBM group; the matching was conducted using the variable ulcer duration. A matching procedure including more variables, for example, sex, frailty level, and ulcer size, would have been preferable but was not possible due to the available register data. The PBM group was recruited from one municipality and represented a specific patient population, potentially limiting the generalizability of the results to younger patients with ALUs. However, frail older adults are an expanding group of patients with ALUs and are frequently excluded from research.

The optimal PBM parameters for different treatment indications, ulcer diagnoses, and patient groups need to be further investigated and understood. Herein, it was not possible to investigate tissue samples and laboratory responses to the PBM treatment, which is a limitation of this study. The patient's clinical picture was carefully monitored until complete healing or other events occurred. Of the 51 patients with ALUs in the PBM group, PBM treatment was disrupted in one patient owing to the limited effects of PBM after 6 weeks; the remaining censored patients showed persistent healing, and treatment was interrupted by non‐ulcer‐related reasons.

## 6 Conclusions

The findings of this study revealed that PBM treatment significantly shortened the healing time of ALUs in frail older adults and generated a higher proportion of healed ulcers than revascularisation or conservative treatment. Thus, PBM was demonstrated to have potential as a promising treatment of ALUs in older adults with frailty, showing to be effective even in the presence of characteristics typically associated with prolonged healing. The non‐invasive nature, low recurrence rate and feasibility for use in home healthcare settings suggest meaningful clinical advantages over more invasive or resource‐intensive treatment approaches.

The findings emphasise the value of long‐term, real‐world studies to further determine the PBM's role in comprehensive wound care strategies for the population of older adults with frailty. Future studies should explore PBM integration into comprehensive geriatric ulcer management protocols, assess cost‐effectiveness, and evaluate long‐term outcomes related to ambulatory status, independence, and healthcare utilisation.

## Author Contributions

M.D. and M.Ö. conceived and created the study methodology. M.D., L.C., M.Ö., M.S.‐E., B.C.B. and Å.A. contributed to the study design. M.D. and M.Ö. performed the data collection and charting, whilst M.D. conducted the analysis with support from L.C. and Å.A. All authors contributed to the discussion and interpretation of the results. M.D. and Å.A. wrote the original draft, which was reviewed and revised by L.C., M.Ö., B.C.B. and M.S.‐E. All authors participated in subsequent revisions and approved the final version of the manuscript, agreeing to be responsible for the accuracy of the presented data.

## Conflicts of Interest

Marianne Degerman participated in courses with Spectro Analytic Irradia AB when the PBM devices were acquired. The other authors declare no conflicts of interest.

## Data Availability

The dataset generated and analysed during the current study is not publicly available because participant consent included restrictions on use of the data due to patients’ privacy concerns. Limited availability is possible. Researchers wishing information should contact Associate Professor Åsa Audulv (asa.audulv@umu.se).
